# Discovery of Novel CRK12 Inhibitors for the Treatment of Human African Trypanosomiasis: An Integrated Computational and Experimental Approach

**DOI:** 10.3390/ph18060778

**Published:** 2025-05-23

**Authors:** Qin Li, Jiayi Luo, Chenggong Fu, Wenqingqing Kang, Lingling Wang, Henry Tong, Zhaorong Lun, Qianqian Zhang, Dehua Lai, Huanxiang Liu

**Affiliations:** 1 Faculty of Applied Sciences, Macao Polytechnic University, Macao SAR, China; qin.li@mpu.edu.mo (Q.L.); p2314521@mpu.edu.mo (C.F.); p1807314@mpu.edu.mo (W.K.); p2212257@mpu.edu.mo (L.W.); henrytong@mpu.edu.mo (H.T.); zhangqq@mpu.edu.mo (Q.Z.); 2 MOE Key Laboratory of Gene Function and Regulation, State Key Laboratory of Biocontrol and Guangdong Provincial Key Laboratory of Aquatic Economic Animals, School of Life Sciences, Sun Yat-Sen University, Guangzhou 510275, China; luojy75@mail2.sysu.edu.cn (J.L.); sslzr@mail.sysu.edu.cn (Z.L.)

**Keywords:** CRK12, human African trypanosomiasis (HAT), molecular docking, virtual screening, neglected tropical disease, drug discovery

## Abstract

**Background:** Human African trypanosomiasis (HAT), caused by *Trypanosoma brucei*, is a neglected tropical disease with limited treatments, highlighting the pressing need for new drugs. Cell division cycle-2-related kinase 12 (CRK12), a pivotal protein involved in the cell cycle regulation of *T. brucei*, has emerged as a promising therapeutic target for HAT, yet effective CRK12 inhibitors remain lacking. **Methods:** An integrated strategy combining computational modeling, virtual screening, molecular dynamics (MD) simulations, and experimental validation was adopted to discover potential inhibitors against CRK12. By using the predicted and refined 3D structure of CRK12 from AlphaFold2 and MD simulation, over 1.5 million compounds were screened based on multiple-scale molecular docking, and 26 compounds were selected for evaluation of biological activity based on anti-*T. brucei* bioassays. Dose–response curves were generated for the most potent inhibitors, and the interaction mechanism between the top four compounds and CRK12 was explored by MD simulations and MM/GBSA binding free energy analysis. **Results:** Of the 26 compounds, six compounds demonstrated sub-micromolar to low-micromolar IC_50_ values (0.85–3.50 µM). The top four hits, F733-0072, F733-0407, L368-0556, and L439-0038, exhibited IC_50_ values of 1.11, 1.97, 0.85, and 1.66 µM, respectively. Binding free energy and energy decomposition analyses identified ILE335, VAL343, PHE430, ALA433, and LEU482 as hotspot residues for compound binding. Hydrogen bonding analysis demonstrated that these compounds can form stable hydrogen bonds with the hinge residue ALA433, ensuring their stable binding within the active site. **Conclusions:** This study establishes a robust and cost-effective pipeline for CRK12 inhibitor discovery, identifying several novel inhibitors demonstrating promising anti-HAT activity. The newly discovered scaffolds exhibit structural diversity distinct from known CRK12 inhibitors, providing valuable lead compounds for anti-trypanosomal drug development.

## 1. Introduction

African trypanosomiasis, also known as sleeping sickness in humans and nagana in animals, is one of the neglected tropical diseases that affects millions of people and livestock across sub-Saharan Africa [1,2]. The causative agent is the vector-borne protozoan parasite *Trypanosoma brucei*, with two subspecies of this parasite, *T. brucei gambiense* and *T. brucei rhodesiense,* being responsible for human infections. *T. brucei gambiense*, predominantly distributed in Western and Central Africa, accounts for approximately 92% of reported cases, typically causing chronic infections [3]. In contrast, *T. brucei rhodesiense*, which is prevalent in Eastern and Southern Africa, is responsible for the remaining 8% of cases and is associated with acute disease progression [3]. Notably, sporadic cases of human African trypanosomiasis (HAT) have been documented in individuals with travel or residence histories in endemic regions, highlighting its broader global relevance [4]. Despite significant advancements in control measures, HAT continues to pose substantial public health challenges, maintaining high mortality rates in affected populations.

Over the past century, only a limited number of drugs have been approved for the treatment of HAT, including pentamidine (for stage 1 of gambiense-HAT), suramin (for stage 1 of gambiense-HAT and rhodesiense-HAT), melarsoprol (for stage 2 of rhodesiense-HAT), eflornithine (for stage 2 of gambiense-HAT), the nifurtimox–eflornithine combination therapy (NECT) (for stage 2 of gambiense-HAT), and the recently approved oral drug fexinidazole (for stage 1 and 2 of gambiense-HAT) [5,6,7]. However, these treatments are associated with significant limitations, such as severe side effects, toxicity, complicated administration regimens, limited availability, and the potential for drug resistance [8,9]. Furthermore, the mechanisms of most of these drugs remain poorly understood, with evidence suggesting they interact with multiple different parasite targets [10]. Therefore, there is an urgent need to identify and develop novel, effective therapeutic options for HAT and offer safer and more effective solutions for managing HAT.

Drug development targeting parasites has focused on critical proteins, such as cell cycle regulators, membrane proteins, and mitochondrial structural proteins [11,12,13]. Among these, the cell division cycle-2-related kinase 12 (CRK12) has already been validated as a promising therapeutic target in *Leishmania* and *Trypanosoma*, with multiple experimental studies confirming its essential role in parasite viability [14,15,16,17]. In 2013, Monnerat et al. demonstrated that depletion of CRK12 in *T. brucei* induces morphological abnormalities, such as enlarged flagellar pockets, and disrupts endocytosis, ultimately leading to parasite death [14]. Subsequently, in 2018, Wyllie et al. provided experimental validation of CRK12 as a therapeutic target for *Leishmania* and reported GSK3186899/DDD853651 as a CRK12 inhibitor with significant antileishmanial activity [15]. More recently, in 2022, Smith et al. designed and synthesized a series of compounds, among which compound 2 showed potent activity against *Trypanosoma congolense* and *Trypanosoma vivax*, with CRK12 identified as the trypanocidal target [16]. Further studies in the same year indicated that compound 2 also exerts anti-trypanosomal effects on *T. brucei* through CRK12 inhibition [17]. Collectively, these findings highlight the key role of CRK12 in *T. brucei* and reinforce its status as a high-priority therapeutic target for HAT.

**Figure 1:**
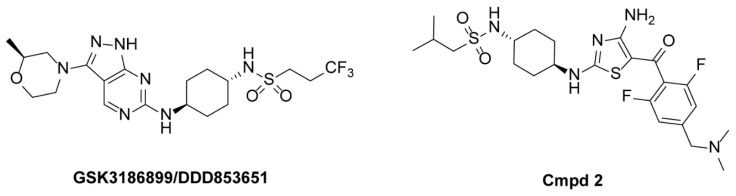
Chemical structures of representative CRK12 inhibitors.

To date, only two classes of small-molecule inhibitors of CRK12 have been identified: pyrazolopyrimidines (GSK3186899/DDD853651) [15] and aminothiazoles (Cmpd 2) [16], with the chemical structures shown in Figure 1. Among these, GSK3186899 is the first potent and selective CRK12 inhibitor to enter clinical trials for the treatment of leishmaniasis [15,18]. However, no CRK12 inhibitors are yet available for clinical treatment. Therefore, the further discovery of efficient and safe CRK12 inhibitors with novel scaffolds to some extent presents promising outcomes.

Potential inhibitors against trypanosomes have been identified using traditional approaches, such as high-throughput screening (HTS) and in vitro inhibitory assays [19,20]. While these methods are essential for validating compound activity, large-scale screening of compound libraries is costly and logistically challenging. In recent years, computational approaches, such as high-throughput virtual screening (HTVS), have emerged as powerful complementary tools [21]. By narrowing the pool of potential candidates in silico, HTVS, in combination with molecular dynamics (MD) simulations, helps prioritize promising compounds for targeted in vitro testing [22,23]. This integrated workflow not only reduces the overall time and resource expenditure but also yields deeper insights into ligand–protein interactions, including dynamic behavior and conformational changes. Consequently, combining computational and experimental strategies offers a cost-effective way to accelerate drug discovery for HAT and to advance the development of innovative therapeutics.

Here, we developed a computational strategy to identify novel CRK12 inhibitors against *T. brucei*. Through structure modeling and molecular docking-based virtual screening, 26 compounds with the potential binding ability to CRK12 were selected. In addition, in vitro assays were conducted to evaluate the inhibitory activity of these compounds against *T. brucei* growth. Finally, six effective inhibitors were identified with IC_50_ values ranging from 0.85 to 3.50 μM. The top four active inhibitors, F733-0072, F733-0407, L368-0556, and L439-0038, demonstrated IC_50_ values of 1.11 µM, 1.97 µM, 0.85 µM, and 1.66 µM, respectively. Molecular dynamics (MD) simulations further elucidated the potential binding mechanisms of these inhibitors, providing detailed insights into their predicted interactions with CRK12. The combination of these computational approaches with experimental validation offers a cost-effective and time-efficient strategy for developing CRK12 inhibitors, advancing the promising discovery of targeted therapies for HAT.

## 2. Results and Discussion

### 2.1. Modelling of the 3D Structure and Active Site of T. brucei CRK12

**Figure 2:**
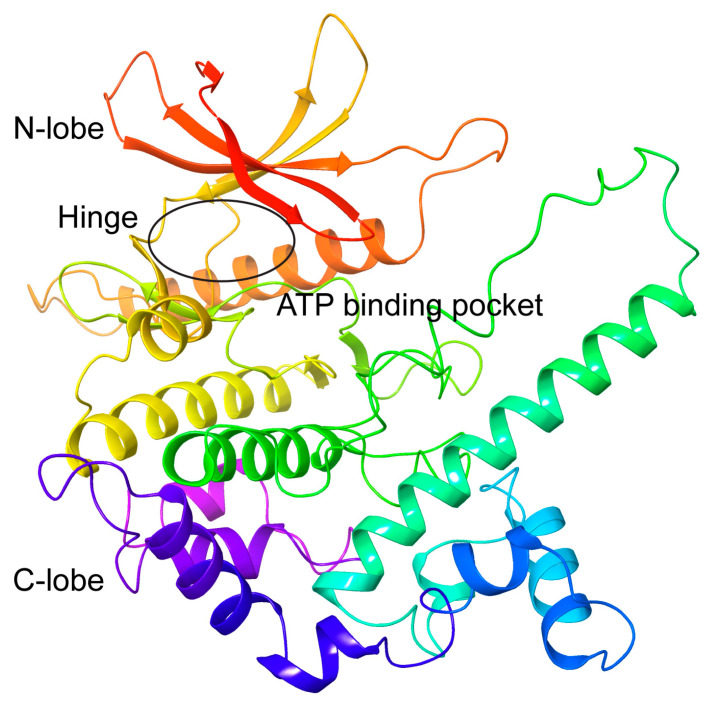
Predicted 3D structure of CRK12 kinase domain by AlphaFold2 (rainbow-colored, N-terminus red to C-terminus purple) and the ATP binding pocket (black circle).

Since the three-dimensional (3D) structure of CRK12 has not yet been solved, we first constructed the 3D structure of *Trypanosoma brucei* CRK12 (TriTrypDB: Tb927.11.12310, UniprotKB: Q382V0) based on AlphaFold2. Structural validation revealed high confidence in the kinase domain, with a mean predicted local distance difference test (pLDDT) score of 82.63 ± 0.82 (scores > 70 indicate reliable backbone predictions). Spatial confidence mapping (Figure S1A) revealed the kinase domain primarily occupies high-confidence regions, with the majority of residues displaying pLDDT scores > 70 (light blue) and significant portions exceeding >90 (dark blue). Consistent with these findings, the predicted aligned error (PAE) matrix (Figure S1B) revealed predominantly low intramolecular positional errors (<5 Å, dark green quadrants) across residues 329–715, further validating the model’s structural reliability. The CRK12 kinase contains a typical ATP-binding domain, comprising a β-sheet-rich N-terminal lobe (N-lobe), an α-helix-dominated C-terminal lobe (C-lobe), and a connecting hinge region (Figure 2). Ramachandran analysis of the predicted structure revealed 88.4% of residues are located in the most favorable regions, 10.4% in additionally allowed regions, and 1.2% in generously allowed regions, with no residues in disallowed regions, indicating the vast majority (>98% total allowed) of backbone conformations are stereochemically optimal (Figure S1C). These results confirm that the AlphaFold2-predicted CRK12 structure is highly reliable and support the AF2-predicted CRK12 model’s suitability for virtual screening and molecular dynamics simulations.

As the structure of CRK12 predicted by AF2 does not bind any small molecules, the potential binding sites should be identified before molecular docking. Here, the active site of CRK12 was identified using the SiteMap [24]. The binding pocket analysis identified five potential binding pockets (Sites 1–5) in the CRK12 AF2 structure, as shown in Figure S2. Upon further evaluation, Site 1, the ATP-binding pocket of the kinase domain, emerges as the most promising pocket. Site 1 exhibits the highest Sitescore of 1.078 and the largest volume of 422.576 Å^3^ (Figure S2). Therefore, Site 1 was selected as the active pocket for subsequent studies.

### 2.2. Refinement of Predicted CRK12 Structure Based on Molecular Dynamics Simulations

The accuracy of virtual screening relies heavily on the reliability of the target protein structure. To improve the quality of the predicted CRK12 structure, a strategy combining molecular docking and MD simulations was employed, incorporating induced-fit effects and eliminating irrational conformations. The known CRK12 inhibitor Cmpd2 (Figure 1), with nanomolar activity against *Trypanosoma brucei* (EC_50_ = 0.96 nM [17]), was docked into the ATP binding pocket identified above. The docking results (Figure S3A) show strong alignment with those reported by Smith [16], where the 2,4-diaminothiazole core of Cmpd2 occupies the adenine region of the ATP-binding pocket, forming critical hydrogen bonds with ALA433 and additional interactions with PRO431, SER436, and ASP493. To further refine the CRK12 structure, a 500 ns MD simulation was performed on the CRK12-Cmpd2 complex structure to optimize the structures and obtain induced-fit effects. Root-mean-square deviation (RMSD) analysis confirmed the stability of the CRK12-Cmpd2 complex throughout the simulation (Figure S3B). Conformational clustering of the MD simulation trajectories was then conducted, and the most representative conformation was extracted as the refined CRK12 structure for subsequent virtual screening.

### 2.3. Assessment of Virtual Screening Performance for the AF2-Predicted CRK12 and MD-Refined CRK12

**Figure 3:**
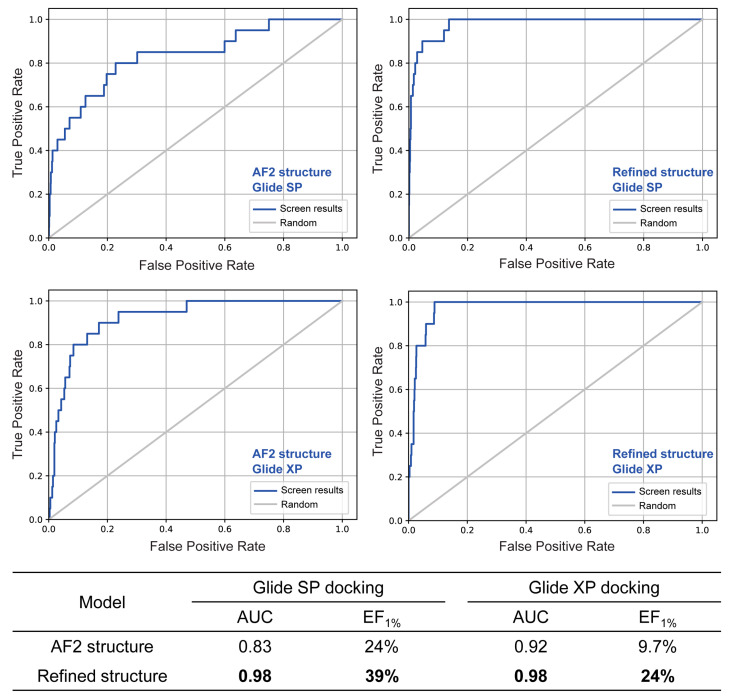
The comparison of virtual screening performance of the AlphaFold2-predicted CRK12 structure and the MD-refined CRK12 structure in glide standard SP and XP docking modes. The table presents AUC and enrichment factors at 1% (EF_1%_). Bold values indicate the best performance for each metric across docking modes, highlighting the superior screening capability of the MD-refined structure.

The AF2-predicted and MD-refined CRK12 structures were compared to evaluate their suitability for virtual screening using datasets containing known active compounds and decoys. Virtual screening was performed with glide in standard-precision (SP) and extra-precision (XP) modes. Its performance was quantified through two critical metrics: (i) global ranking accuracy via the area under the receiver operating characteristic curve (AUC), and (ii) early enrichment capability via the enrichment factor at the top 1% of ranked compounds (EF_1%_) [25]. AUC reflects overall enrichment performance by measuring the model’s ability to rank true actives above inactives across the dataset. EF_1%_ captures early enrichment efficiency, which is critical for practical applications, as only top-ranked compounds typically advance to experimental validation. As shown in Figure 3, the MD-refined structure demonstrated significant improvements in both performance metrics. Specifically, the refined structure achieved near-perfect global discrimination with AUC values of 0.98 in both SP and XP modes (15 and 6 percentage points increases over the unrefined AF2 predicted structure, respectively). Most notably, EF_1%_ values increased by 15 and 14 percentage points in SP (24% → 39%) and XP (9.7% → 23.7%) modes, respectively. These substantial improvements demonstrate that MD refinement effectively corrects conformational biases inherent in the AlphaFold2 predicted structure, significantly enhancing docking reliability. The marked boost in early enrichment performance is particularly crucial for hit identification in virtual screening campaigns. These findings establish MD refinement as an essential step for optimizing AF2-predicted structures in structure-based drug discovery pipelines and strongly support the use of the MD-refined CRK12 model for downstream multi-stage virtual screening.

### 2.4. Results of Structure-Based Virtual Screening

**Figure 4:**
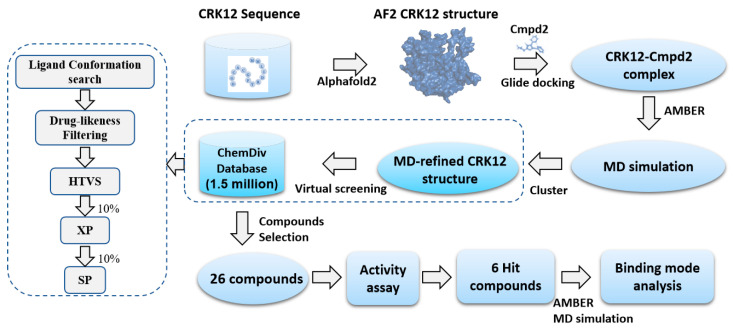
Schematic workflow of structure-based virtual screening.

The whole structure-based virtual screening (SBVS) process used in this study is shown in Figure 4. Based on the MD-refined CRK12 structure, molecular docking-based virtual screening was performed to identify potential inhibitors targeting CRK12 from the Chemdiv database. The virtual screening workflow employed three sequential precision modes: high-throughput virtual screening (HTVS), standard precision (SP), and extra precision (XP). This multi-stage approach successfully identified thousands of high-potential compounds for further investigation. To ensure structural diversity, K-means clustering was applied based on binary fingerprints, categorizing the compounds into hundreds of distinct clusters. This strategy enhanced the chemical diversity of the candidate compounds, promoting the selection of molecules with varied backbone scaffolds. The compounds in each cluster were then carefully evaluated, focusing on lower docking scores, lower molecular weights, and the propensity to form hydrogen bonds with the kinase hinge residue ALA433. Based on these criteria, we finally chose 26 compounds as the most promising candidate compounds for subsequent experimental validation. The physicochemical properties and docking results for these compounds are provided in Table S1.

### 2.5. In Vitro Inhibitory Activity of the 26 Selected Compounds Against T. brucei

**Figure 5:**
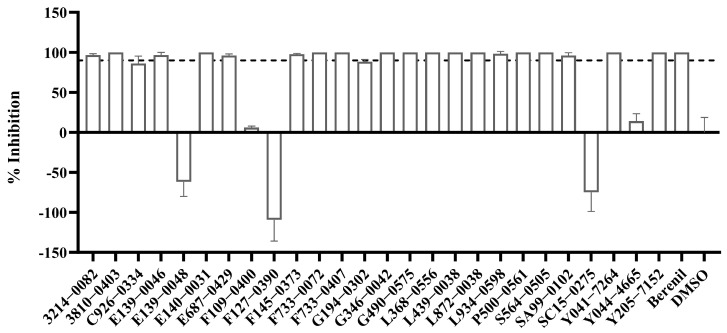
In vitro inhibitory activities of all 26 selected compounds at a concentration of 50 μM against *T. brucei*. The dashed line indicates the 90% inhibition threshold. Berenil was used as a positive control.

**Figure 6:**
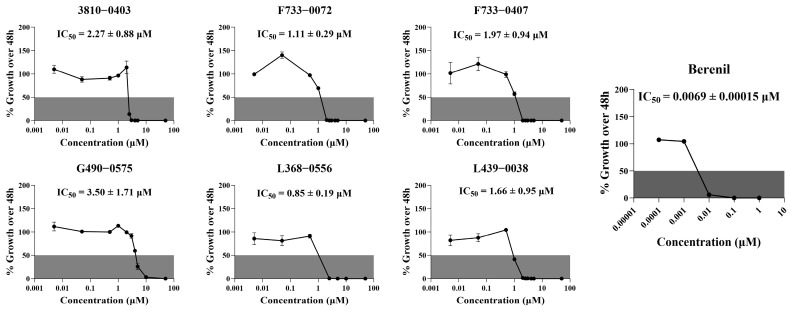
Dose–response curves of 6 active compounds against *T. brucei* growth over 48 h. The IC_50_ values were derived from three independent in vitro inhibitory assays and are presented as mean ± SEM.

**Figure 7:**
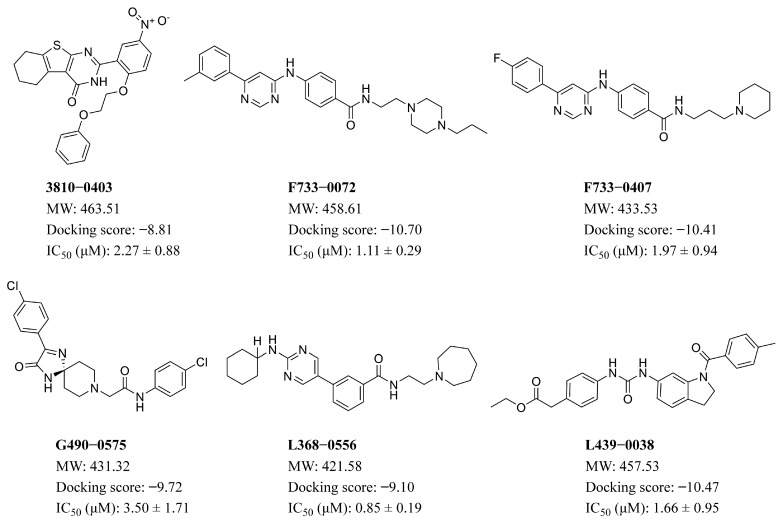
2D chemical structures along with their docking scores and IC_50_ values of 6 hit compounds identified by in vitro inhibition assays against *T. brucei* growth.

From the virtual screening process, 26 compounds were initially selected as potential candidates for further evaluation of their biological activity against *Trypanosoma brucei*. The compounds were first evaluated for the in vitro anti-trypanocidal activity at a concentration of 50 µM. Notably, 19 of 26 compounds exhibited over 90% inhibition rate, indicating a high hit rate and demonstrating the robustness and efficiency of our virtual screening approach. Among them, 13 compounds achieved complete (100%) inhibition against *T. brucei* bloodstream forms (BSF) (Figure 5). We then performed dose-response experiments on the six most potent compounds, which all showed 100% inhibition in initial testing. The results confirmed their strong activity, with IC₅₀ values below 5 µM (Figure 6 and Figure 7). Among these, the top four highly active compounds, F733-0072, F733-0407, L368-0556, and L439-0038, exhibited IC_50_ values of 1.11 µM, 1.97 µM, 0.85 µM, and 1.66 µM, respectively. Berenil, a well-known anti-trypanosome drug, was used as the positive control (IC_50_ = 0.0069 µM in this study; Figure 6). Notably, the top four compounds also showed favorable docking scores (Figure 7), suggesting a consistent correlation between computational predictions and in vitro potency. Owing to their promising potency, these four highly active compounds were selected for MD simulations to further investigate their binding mechanisms with CRK12.

### 2.6. Molecular Dynamics Simulations

**Figure 8:**
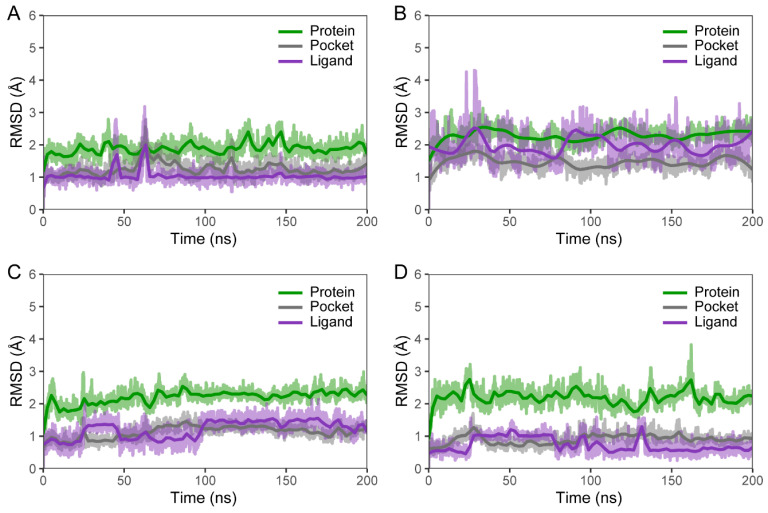
The RMSD of the backbone atoms of the CRK12 protein, the heavy atoms of the pocket, and the heavy atoms of the ligands for complexes with (**A**) F733-0072, (**B**) F733-0407, (**C**) L368-0556, and (**D**) L439-0038.

To explore the potential binding mechanism of the four highly active inhibitors mentioned above (F733-0072, F733-0407, L368-0556, and L439-0038), 200 ns MD simulations were performed for four protein–ligand complex systems. The initial binding poses used for these simulations were obtained through molecular docking. The stability of each system was evaluated by measuring the root mean square deviation (RMSD) of the backbone atoms of the CRK12, the binding pocket, and the heavy atoms of the ligand throughout the simulation period. As shown in Figure 8, the RMSD plots indicate that the protein, binding pocket, and ligands in all four systems remained stable throughout the simulation, with average fluctuations ranging from 0.5 to 2 Å. Notably, all systems reached convergence after approximately 150 ns, implying stable binding of the ligands to CRK12. Given these observations, the last 50 ns of the equilibrium trajectory for each system was used for subsequent analysis.

### 2.7. Binding Free Energy Calculation and Hot Spot Residue Identification

**Figure 9:**
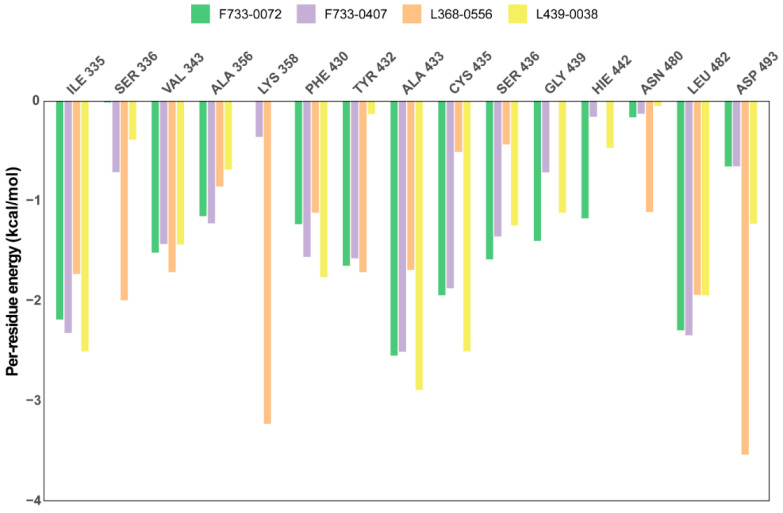
The energy contributions of key residues responsible for the binding of four inhibitors: F733-0072, F733-0407, L368-0556, and L439-0038.

To estimate the binding affinities of four inhibitors to CRK12, the molecular mechanics generalized Born surface area (MM-GBSA) method was used to calculate the binding free energy between ligands and protein. As summarized in Table 1, the calculated ${∆G}_{bind}$ values for F733-0072, F733-0407, L368-0556, and L439-0038 are −48.38 kcal/mol, −44.55 kcal/mol, −54.64 kcal/mol, and −45.64 kcal/mol, respectively. These results suggest the strong binding affinities of the inhibitors, further validating the potency of the identified compounds. To identify the key residues involved in ligand–protein binding, per-residue decomposition analysis was performed. Residues with significant binding contributions (>1.0 kcal/mol) are shown in Figure 9. The identified key residues include ILE335, ILE336, VAL343, ALA356, LYS358, PHE430, TYR432, ALA433, CYS435, SER436, GLY439, HIE442, ASN480, LEU482, and ASP493. Among these, ILE335, VAL343, PHE430, ALA433, and LEU482 demonstrated consistently large binding contributions across all four complexes, suggesting their potential as hotspot residues for CRK12 inhibitor development. These residues displayed low conformational flexibility in RMSF analysis (Figure S4), suggesting ligand-induced stabilization and further supporting their critical role in binding interactions. Two residues, TYR432 and ALA433, are located in the hinge region, which also demonstrates the important role of the hinge region in the binding of CRK12 inhibitors. Notably, LYS358 and ASP493 showed extremely high energy contributions (>3 kcal/mol) to the binding of L368-0556 (Figure 9), underscoring their importance in stabilizing this highly potent inhibitor.

### 2.8. Hydrogen Bond Analysis Between Four Hits and CRK12

We first analyzed hydrogen bond formation between the four hit compounds and CRK12 during the 200 ns MD simulations. As shown in Figure S5, each system maintained 2–5 stable hydrogen bonds throughout the simulation trajectory. To further evaluate their binding contributions, we calculated hydrogen-bond occupancies during the final 50 ns of simulation (Table 2). A common feature across all inhibitors was the formation of strong hydrogen bonds with the hinge residue ALA433, a hallmark interaction of ATP-competitive kinase inhibitors. These hydrogen bonds, involving ALA433@O and ALA433@N-H groups, exhibited high occupancy rates, emphasizing the importance of ALA433 in anchoring ligands within the ATP-binding site. A previous study by Smith et al. also demonstrated that this residue is critical for ligand binding [16]. Our MM-GBSA calculations further support this finding, with ALA433 demonstrating substantial binding contributions (≥1.5 kcal/mol) across all four complexes. These consistent results establish ALA433 as a key anchoring site for CRK12 inhibitor design. F733-0072 showed two stable hydrogen bonds with ALA433, with a high occupancy rate of 96.26% (ALA433@N-H) and 99.81% (ALA433@O), respectively. Similarly, F733-0407 also displayed two stable hydrogen bonds with ALA433, with an occupancy rate of 92.75% (ALA433@N-H) and 99.74% (ALA433@O). Although L368-0556 exhibited comparatively lower occupancy rates with ALA433 (87.48% for ALA433@N-H and 68.75% for ALA433@O), it compensated with additional strong interactions. Notably, L368-0556 formed a stable hydrogen bond with ASP493, a key residue in the DFG motif (occupancy rate: 80.09%), which accounted for the high energy contribution of residue ASP493 to the binding. Additionally, L368-0556 also established three other hydrogen bonds with LYS358 (LYS358@NZ-HZ1, LYS358@NZ-HZ2, and LYS358@NZ-HZ3), totaling an occupancy rate of 95.11%, further enhancing its binding stability. L439-0038 exhibited stable hydrogen bonding with ALA433 (occupancy rates: 99.55% and 95.34%) and additional interactions with ASP493 (95.09%) and SER436 (91.45% and 69.64%). These interactions underscore the significance of hinge residues (e.g., ALA433) and DFG motif residues (e.g., ASP493) in stabilizing ligand binding and provide valuable insights into the design of potent CRK12 inhibitors.

### 2.9. Binding Mode Analysis Between the Four Hits and CRK12

**Figure 10:**
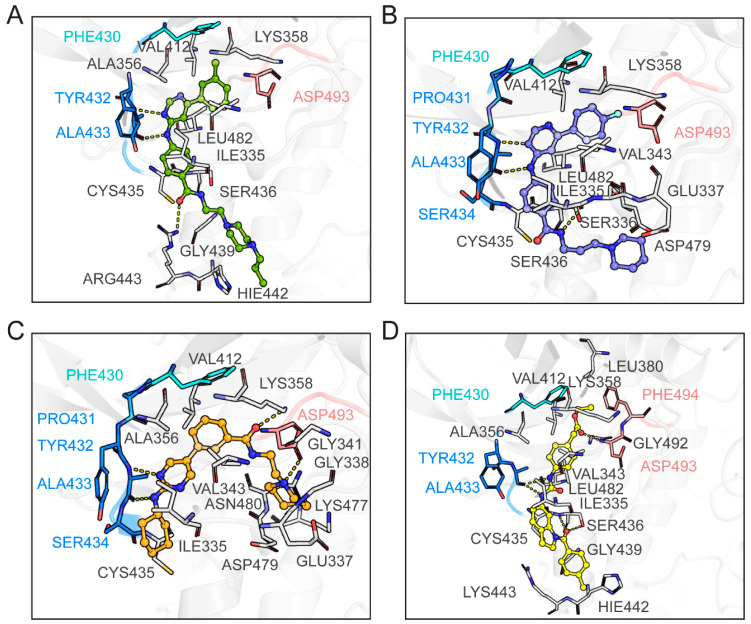
The binding mode between the four compounds and CRK12. The inhibitors (**A**) F733-0072, (**B**) F733-0407, (**C**) L368-0556, and (**D**) L439-0038 are shown as green, slate, orange, and yellow sticks, respectively. The CRK12 protein is shown as the gray cartoon, and the key residues are shown as the gray sticks, with gatekeeper residue colored in cyan, hinge residues colored in blue, and ASP-PHE-GLY (DFG) motif colored in lotus pink. The dotted yellow lines represent hydrogen bonds.

To identify representative binding modes, we performed cluster analysis on the final 50 ns of each simulation trajectory and extracted the most populated conformations (Figure 10). The simulations revealed that all four ligands adopt a narrow, extended conformation within the CRK12 binding pocket. Each compound appears to position its nitrogen heterocycle or urea group in the adenine region of the ATP-binding site, consistently forming hydrogen bonds with the hinge residue ALA433. Specifically, F733-0072, F733-0407, and L368-0556 may rely on the aminopyrimidine cores for hinge binding, while L439-0038 employs a urea group to interact with ALA433 (Figure 10). While the aminopyrimidine scaffold is a common hinge binder in kinase inhibitors [26], the urea group potentially represents an alternative scaffold for targeting kinases. These interactions with the hinge region are critical for anchoring the ligands and mimicking ATP binding. Beyond the hinge region, structural analysis reveals all ligands exploit a conserved hydrophobic subpocket beneath the P-loop through distinct aromatic interactions: terminal 3-methylphenyl (F733-0072); terminal 4-fluorophenyl (F733-0407); central benzene rings (L368-0556, L439-0038). Variations in ligand structures could lead to distinct binding characteristics, enabling additional interactions with CRK12 residues. For instance, F733-0072 seems to form a hydrogen bond with the C-lobe residue ARG443 through its amide linker, while F733-0407 appears to engage the N-lobe residue ILE335 in a similar manner (Figure 10A,B). L368-0556 and L439-0038 extend their interactions into additional regions, particularly the DFG motif. L368-0556 adopts a U-shaped conformation that may directly interact with ASP493 of the DFG motif and form another hydrogen bond with the N-lobe residue LYS358 (Figure 10C). Conversely, L439-0038 appears to occupy a deeper hydrophobic pocket near the gatekeeper using its terminal ethyl ester “handle”, stabilizing itself with a hydrogen bond to ASP493 and an additional hydrogen bond with the C-lobe residue SER436 (Figure 10D). The extent of solvent exposure may also differ among the inhibitors. F733-0072, F733-0407, and L439-0038 exhibit relatively long, solvent-exposed tails, such as the 2-(4-propylpiperazin-1-yl) ethyl group of F733-0072, the 3-(1-piperidinyl) propyl group of F733-0407, and the 4-methylphenyl group of L439-0038. In contrast, L368-0556 appears to be more deeply buried within the pocket, with only its 2-azepan-1-yl-ethyl group and cyclohexyl group minimally exposed to solvent. Overall, these proposed binding modes and interaction profiles may provide valuable insights for the rational design of new CRK12 inhibitors.

In summary, this study developed an integrated structure-based lead discovery workflow by combining target structure prediction and refinement, multi-scale docking, and biological assays. Based on this pipeline, 26 compounds were selected for biological activity evaluation, and 19 exhibited over 90% inhibition rate, indicating a high hit rate and demonstrating the robustness and efficiency of our virtual screening workflow. Among the identified hits, six compounds exhibited low-micromolar potency against human African trypanosomiasis. The interaction mechanism of the top four candidates with CRK12 was further explored through molecular dynamics simulations and MM-GBSA binding free energy calculations. Structural analysis revealed critical interactions with hinge residue ALA433 and identified key hotspot residues (ILE335, VAL343, PHE430, LEU482) of CRK12, providing a clear framework for subsequent lead optimization. These findings not only expand the chemical space of CRK12 inhibitors but also deliver promising lead compounds for the development of next-generation therapeutics against human African trypanosomiasis.

## 3. Materials and Methods

### 3.1. 3D-Structure Prediction and Binding Site Identification of Trypanosoma brucei CRK12

The 3D structure of *Trypanosoma brucei* CRK12 was predicted by AlphaFold2 (https://alphafold.ebi.ac.uk, accessed on 22 September 2022). The predicted structure was verified via a Ramachandran plot by using PROCHECK [27]. The active site of CRK12 was identified using the SiteMap in Schrödinger (Release 2022, Schrödinger, LLC, New York, NY, USA) and evaluated using different physicochemical descriptors such as Sitescore, Dscore, and Volume.

### 3.2. Molecular Docking-Based Virtual Screening

A combining strategy was used to refine the predicted protein structure as below. First, we generated a ligand-induced *holo* conformation of CRK12 by docking the known ligand into the AlphaFold2-predicted structure. Subsequently, the ligand-bound complex underwent 500 ns of molecular dynamics (MD) simulation to sample conformational space, from which a representative structure was selected for virtual screening. Prior to performing the large-scale virtual screening, the virtual screening performance was assessed based on the active dataset (23 known active CRK12 compounds from references [15,16]) and a decoy dataset (1:50 ratio) generated via the DUD-E database [28] (http://dude.docking.org, accessed on 28 October 2022). We evaluated virtual screening performance using receiver operating characteristic (ROC) analysis, quantifying both the area under the curve (AUC) and enrichment factors (EF) to assess the model’s ability to distinguish active ligands from inactive compounds [29]. The whole molecular docking-based virtual screening process was performed by Schrödinger 2022.

All ligands (active datasets and the compounds from the ChemDiv, San Diego, CA, USA) were prepared using the LigPrep module, including 2D to 3D structure conversion and conformation search. The prepared ligands were first filtered using QikProp and Lipinski’s rule of five [30] to remove the molecules with reactive functional groups. The 3D protein structure was prepared using the protein preparation wizard in the Schrodinger suite, including the addition of hydrogen atoms and missing side chain atoms, while the protein protonation state was assigned using PROPKA at pH 7.0 ± 2.0. Finally, the entire structure was minimized using the OPLS-2005 force field with the heavy atom RMSD convergence of 0.3 Å. For the docking of all ligands, the receptor grid generation module was used to generate the docking grid file.

The virtual screening workflow employed a tiered docking approach using the ChemDiv compound library, proceeding through three successive precision modes: (1) HTVS stage—high-throughput virtual screening of the entire library, with the top 10% highest-scoring compounds advanced to SP docking; (2) SP stage—standard precision docking of HTVS hits, retaining the top 10% performers for XP docking; (3) XP stage—extra precision evaluation, from which the top 4000 compounds were selected for further analysis. For each molecule, only the top one binding pose was saved. The obtained 4000 molecules were then clustered into 100 groups using the K-means algorithm in Canvas to select the compounds with diverse scaffolds. Within each group, compounds with the lowest docking scores, best MM/GBSA scores, and small molecular weights were prioritized for selection. By visual inspection of the binding modes of the CRK12 protein with ligands, 26 compounds were selected and purchased from TargetMol (Boston, MA, USA, https://www.tsbiochem.com) for further in vitro inhibitory assays.

### 3.3. Molecular Dynamics Simulation

MD simulations were performed on the complexes of CRK12 with the known active compound Cmpd2 and the identified representative compounds to obtain the refined CRK12 complex structure and explore the binding mechanism of these compounds, respectively. All initial structures of each system for MD simulations were obtained from molecular docking. Molecular geometries of Cmpd2 and the four compounds were optimized using Gaussian 16 [31] at the HF/6-31G* level with singlet spin multiplicity (multiplicity = (1)) and tight convergence criteria. Optimizations were considered converged when simultaneously satisfying the following: (1) maximum force ≤ 4.5 × 10⁻^4^ a.u.; (2) RMS force ≤ 3.0 × 10⁻^4^ a.u.; (3) maximum displacement ≤ 1.8 × 10⁻^3^ a.u.; (4) RMS displacement ≤ 1.2 × 10⁻^3^ a.u. Then, the restrained electrostatic potential (RESP) charges were fitted by the antechamber and parmchk modules in the AMBER20 package (University of California, San Francisco, CA, USA). The RESP atomic charges of Cmpd2 are given in Table S2 as an example. The force field parameters for each compound were generated by the antechamber program. Then, tleap module in AMBER20 was used to generate the topology and coordinate files for each system. The protein and ligand were described using AMBER ff14SB [32] and GAFF [33] force fields, respectively. Then, each system was neutralized with Na^+^ or Cl^−^ ions and immersed in a TIP3P water model [34], with the water box extending 10 Å beyond all solute atoms in each dimension. In order to avoid boundary effects, the periodic boundary condition (PBC) was also applied during the simulation.

All MD simulations were performed in the AMBER20 package. Initially, energy minimization was carried out by the steepest descent method for the first 2500 steps and the conjugated gradient method for the subsequent 2500 steps. Afterwards, each system was heated up from 0 K to 300 K under the NVT ensemble using a Langevin thermostat while restraining the complex with a 2.0 kcal/(mol·Å^2^) force constant. Thereafter, each system was equilibrated with decreasing restraints from 2.0 to 0 kcal/(mol·Å^2^) in the NPT ensemble at 300 K. Finally, the production MD simulations were carried out for each system in the NPT ensemble at 300 K without any restraints. In addition, long-range electrostatic interactions were treated by the particle mesh Ewald (PME) algorithm [35], and the covalent bonds involving hydrogen atoms were limited by the SHAKE algorithm [36].

### 3.4. Molecular Mechanics Generalized-Born Surface Area Calculation

Assessing the binding affinity of the ligands to the protein is important in elucidating molecular interactions. In this study, the molecular mechanics-generalized Born surface area (MM-GBSA) method was used to calculate the binding free energy between ligand and protein, which has been widely used in many studies [37,38,39]. The basic principle can be described by the following equations:

$${∆G}_{\mathrm{bind}}={∆G}_{\mathrm{complex}\;}-(∆G_{\mathrm{receptor}}+{∆G}_{\mathrm{ligand}})$$

$${∆G}_{\mathrm{bind}}=∆H-T∆S=∆E_{\mathrm{MM}}+{∆G}_{\mathrm{SOLV}}-T∆S$$

$${∆G}_{\mathrm{GAS}}={\;∆E_{\mathrm{MM}}=∆E_{\mathrm{INT}}+∆E}_{\mathrm{VDW}}+{∆E}_{\mathrm{ELE}}$$

$${∆G}_{\mathrm{SOLV}}={∆E}_{\mathrm{GB}}+{∆E}_{\mathrm{SURF}}$$

where ${∆G}_{\mathrm{bind}}$ is the binding free energy, which can be estimated by the free energies of complex ($∆G_{\mathrm{complex}})$, protein ($∆G_{\mathrm{receptor}}$), and ligand (${∆G}_{\mathrm{ligand}})$, respectively. $∆E_{\mathrm{MM}}$ is the gas-phase interaction energy change between protein and ligand. The change of solvation-free energy (${∆G}_{\mathrm{SOLV}})$ can be calculated using the GB equation [40], including the polar solvation-free energy (ΔG_GB_) and nonpolar solvation-free energy (ΔG_SURF_). A water probe radius of 1.4 Å was used to estimate G_SURF_ from solvent-accessible surface area [41]. The surface tension constant (γ) was set to 0.0072 kcal/(mol·Å^2^), and the nonpolar contribution term *β* was set to 0 [42]. To determine the contribution of individual residues to the total binding free energy between ligand and CRK12, the calculated binding free energy was decomposed into each residue.

### 3.5. Trypanosome Cultures and Inhibitory Assays In Vitro

The origin of the pleomorphic *Trypanosoma brucei* AnTat1.1 strain (EATRO1125) used in this study has been previously described [43]. *T. brucei* bloodstream form (BSF) trypanosomes were maintained at a density of below 1 × 10^6^ cells/mL in Hirumi-modified Iscove’s medium 9 (HMI-9) [44]. For the inhibitory assay, parasites harvested from the log phase growth (5 to 8 ×10^5^ cells/mL) were prepared for compound testing. Tested compounds dissolved in DMSO were then added to the cultures, ensuring a final DMSO concentration of 0.1% in the assay medium. Parasites were cultured at an initial density of 5 ×10^3^ cells/mL in 12-well plates with a final volume of 3 mL per well. Plates were incubated at 37 °C in a humidified incubator with 5% CO_2_ for 48 h. After incubation, the number of motile parasites was assessed using a Neubauer chamber (Marienfeld) under a light microscope (LEICA DM500). The group containing 0.1% DMSO without the addition of test compounds was used as the 0% inhibition control. Each biological replicate experiment contained the well-studied compound Berenil (diminazene aceturate, for animal African trypanosomiasis) as the positive group to confirm the sensitivity of *T. brucei* to antitrypanosomal agents. All the compounds were tested first at 50 µM concentration to ensure a sufficient threshold for detecting potential activity, and those that showed strong activity at this level were subsequently subjected to a 10-fold serial dilution for further inhibitory assays. Results from this gradient dilution approach enabled later inhibitory assays at various concentrations to construct detailed dose–response curves, which in turn allowed for the precise determination of the IC_50_ values. The IC_50_ values derived from three independent biological replicate experiments were calculated through a nonlinear regression analysis using GraphPad Prism 10 software.

## 4. Conclusions

This work presents a structure-based drug discovery pipeline that successfully identified novel inhibitors of *T. brucei* CRK12 as potential treatments for HAT. By refining the AlphaFold2-predicted CRK12 structure using MD simulations, the performance of virtual screening was highly enhanced. Through a three-stage virtual screening process, 26 candidate compounds were selected for further experimental evaluation. Among these, six compounds exhibited strong inhibitory activity. The interaction mechanism between the top four compounds and CRK12 was further explored by molecular dynamics simulations. The MD simulations and binding energy analyses revealed critical interactions with hinge residue ALA433 and other hotspot residues (e.g., ILE335, VAL343, PHE430, LEU482), which explain the high potency of these inhibitors and guide future structural optimization. In conclusion, this study establishes a robust and cost-effective pipeline for CRK12 inhibitor discovery, identifying multiple novel chemotypes demonstrating promising anti-HAT activity. The newly discovered scaffolds exhibit structural diversity distinct from known CRK12 inhibitors, providing valuable lead compounds for anti-trypanosomal drug development. Building on these findings, our future work will focus on the following: (1) systematic structure–activity relationship (SAR) optimization to enhance potency; (2) rational modification to improve pharmacokinetic properties and selectivity; (3) comprehensive in vitro and in vivo evaluation of candidate compounds. These efforts will facilitate the translation of these molecular scaffolds into potential clinical candidates for human African trypanosomiasis treatment.

## Figures and Tables

**Table 1:** The binding free energies of F733-0072, F733-0407, L368-0556, and L439-0038 with CRK12 using the MM-GBSA method (kcal/mol). Results are shown as mean ± SEM.

Compound ID	F733-0072	F733-0407	L368-0556	L439-0038
${∆E}_{VDW}$	−53.72 ± 0.09	−52.87 ± 0.11	−53.60 ± 0.11	−54.07 ± 0.11
${∆E}_{ELE}$	103.86 ± 0.33	95.42 ± 0.73	20.58 ± 0.38	−20.70 ± 0.17
${∆E}_{GB}$	−91.93 ± 0.32	−80.57 ± 0.70	−14.48 ± 0.34	36.07 ± 0.12
${∆E}_{SURF}$	−6.59 ± 0.01	−6.53 ± 0.01	−7.15 ± 0.01	−6.95 ± 0.01
${∆G}_{GAS}$	50.14 ± 0.34	42.55 ± 0.75	−33.01 ± 0.40	−74.76 ± 0.20
${∆G}_{SOLV}$	−98.52 ± 0.32	−87.10 ± 0.69	−21.63 ± 0.34	29.12 ± 0.12
${∆G}_{bind}$	−48.38 ± 0.12	−44.55 ± 0.12	−54.64 ± 0.15	−45.64 ± 0.13
IC_50_ (µM)	1.11 ± 0.29	1.97 ± 0.94	0.85 ± 0.19	1.66 ± 0.95

**Table 2:** The hydrogen bond occupancy between CRK12 and the four compounds during the simulation. The hydrogen bonds formed with the hinge residue ALA433 are in bold.

Compound ID	Donor	Acceptor	Distance (Å)	Angle (°)	Occupancy (%)
F733-0072	**LIGAND@N5-H3**	**ALA433@O**	2.90	156.69	99.81
	**ALA433@N-H**	**LIGAND@N1**	3.04	150.22	96.26
	ARG443@NH2-HH22	LIGAND@O1	2.94	143.20	30.69
	LIGAND@N6-H5	SER436@OG	3.26	144.88	28.37
	ARG443@NH1-HH12	LIGAND@O1	2.99	144.52	17.41
F733-0407	**LIGAND@N4-H3**	**ALA433@O**	2.93	158.34	99.74
	**ALA433@N-H**	**LIGAND@N1**	3.01	146.45	92.75
	LIGAND@N5-H4	SER436@OG	3.23	143.18	23.41
	LIGAND@N5-H4	ILE335@O	3.03	147.91	19.60
L368-0556	**ALA433@N-H**	**LIGAND@N1**	3.13	156.48	87.48
	LIGAND@N3-H36	ASP493@OD1	2.74	165.06	80.09
	**LIGAND@N4-H2**	**ALA433@O**	3.04	149.30	68.75
	LIGAND@N5-H5	SER336@OG	3.17	146.78	44.38
	LYS358@NZ-HZ1	LIGAND@O1	2.82	150.06	33.47
	LYS358@NZ-HZ2	LIGAND@O1	2.82	149.50	33.38
	LYS358@NZ-HZ3	LIGAND@O1	2.82	149.72	28.26
L439-0038	**LIGAND@N2-H4**	**ALA433@O**	2.88	159.00	99.55
	**LIGAND@N3-H5**	**ALA433@O**	3.06	151.9	95.34
	ASP493@N-H	LIGAND@O3	2.93	158.29	95.09
	SER436@N-H	LIGAND@O1	2.98	152.47	91.45
	SER436@OG-HG	LIGAND@O1	2.80	161.36	69.64

## Data Availability

Data is contained within the article or Supplementary Materials.

## Supplementary Materials

The following supporting information can be downloaded at: https://www.mdpi.com/article/10.3390/ph18060778/s1, Figure S1: The AlphaFold2 predicted structure of *T. brucei* CRK12. Figure S2: Binding site prediction of *T. brucei* CRK12 kinase domain using SiteMap module. Figure S3: The binding conformation of Cmpd2 in the *T. brucei* CRK12 pocket and the overall structural stability of the CRK12-Cmpd2 complex. Figure S4: Root mean square fluctuation (RMSF) of Cα atoms in CRK12 for four systems. Figure S5: The monitoring of the total number of hydrogen bonds between CRK12 and four hit compounds (A) F733-0072, (B) F733-0407, (C) L368-0556, and (D) L439-0038 during 200 ns MD simulations. Table S1: Physicochemical properties of the compounds derived from the virtual screening. Table S2: The coordinates (Å) and RESP atomic charges for the optimized geometry of ligand cmpd2 obtained at the HF/6-31G level.
