# Supplementary material for: Discovery of Novel CRK12 Inhibitors for the Treatment of Human African Trypanosomiasis: An Integrated Computational and Experimental Approach
Source: Pharmaceuticals (Basel). 2025 May 23;18(6):778. doi: 10.3390/ph18060778 (PMC12196357; doi:10.3390/ph18060778)
Supplement: Supplementary file 1 [file pharmaceuticals-18-00778-s001.zip › pharmaceuticals-3618918-supplementary.pdf]

Supplementary Information

Discovery of Novel CRK12 Inhibitors for the Treatment of Human African Trypanosomiasis: An Integrated Computational and Experimental Approach

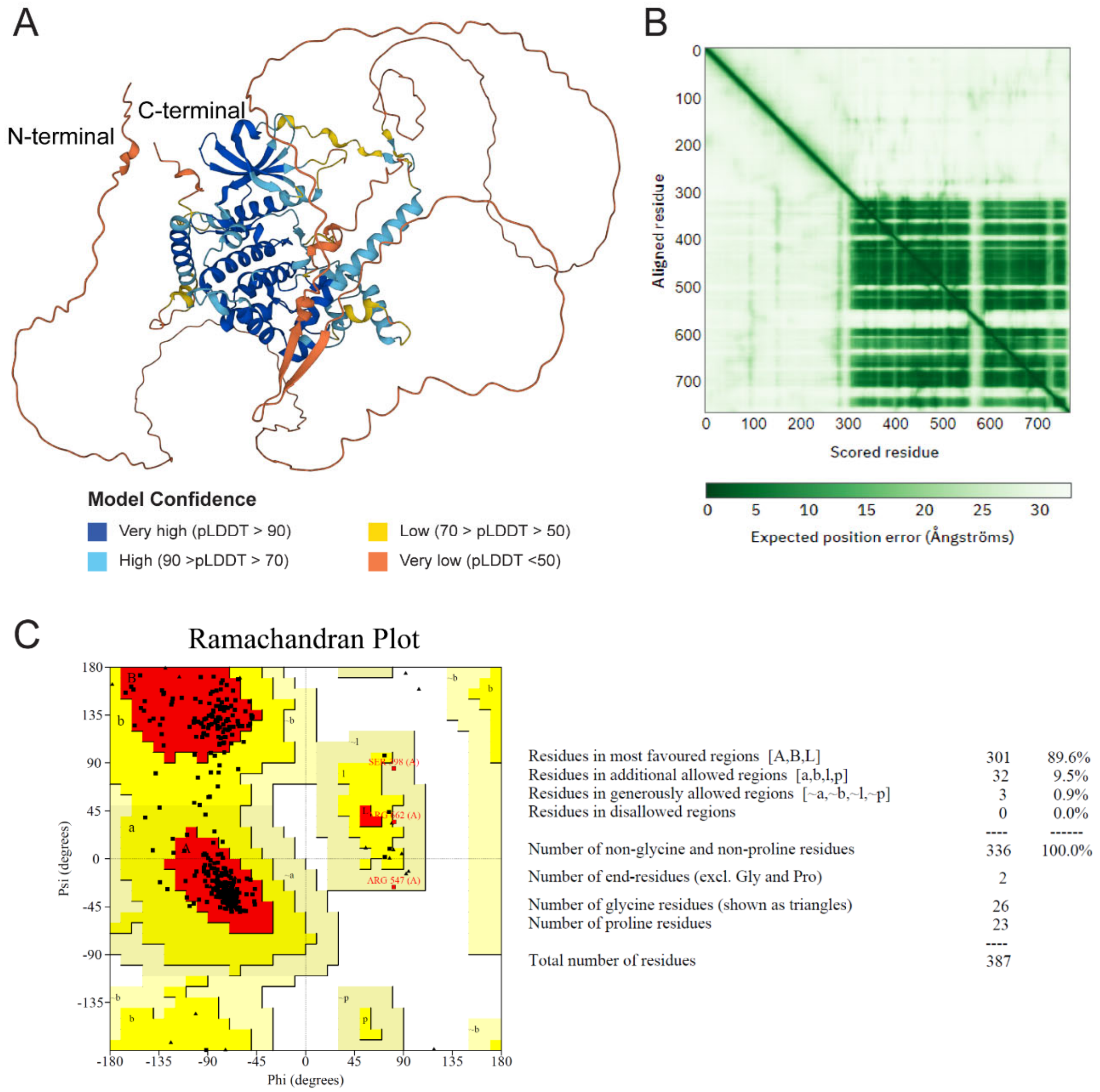

Figure S1. The AlphaFold2 predicted structure of *T. brucei* CRK12. (**A**) The AlphaFold2 predicted the full-length structure of CRK12. Different colors indicate AlphaFold2's level of confidence in its prediction. (**B**) Predicted aligned error (PAE) plot, showing regions of high confidence (dark green) and low confidence (pale green) for the predicted structure. (**C**) Ramachandran plot of the modeled CRK12 kinase domain.

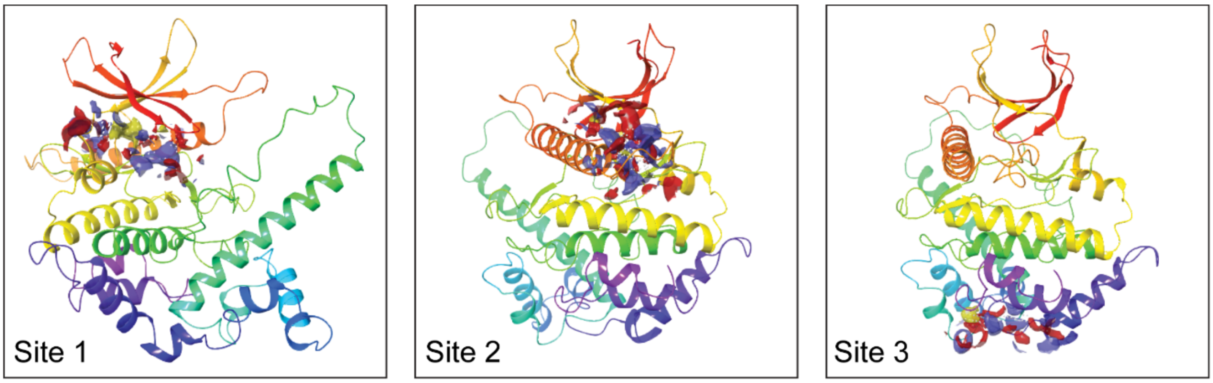

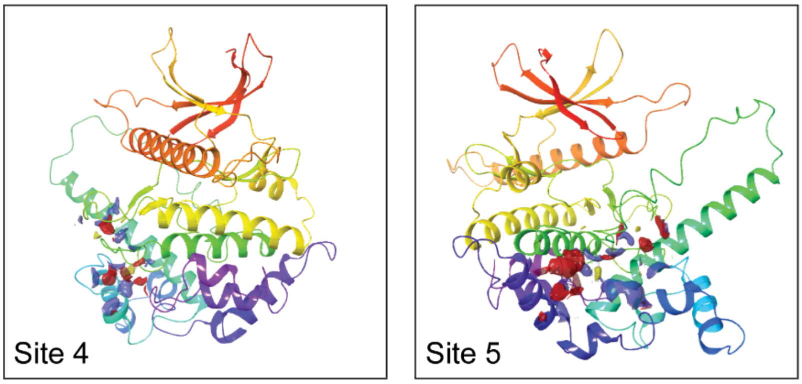

Sites	Site score	Dscore	Volume (Å^3)
Site 1	1.078	1.05	422.576
Site 2	0.938	0.964	285.719
Site 3	0.836	0.818	168.07
Site 4	0.751	0.751	142.345
Site 5	0.805	0.668	210.259

Figure S2. Binding site prediction of *T. brucei* CRK12 kinase domain using SiteMap module. Five active sites 1–5 are identified. Site 1 coincides with the ATP binding site.

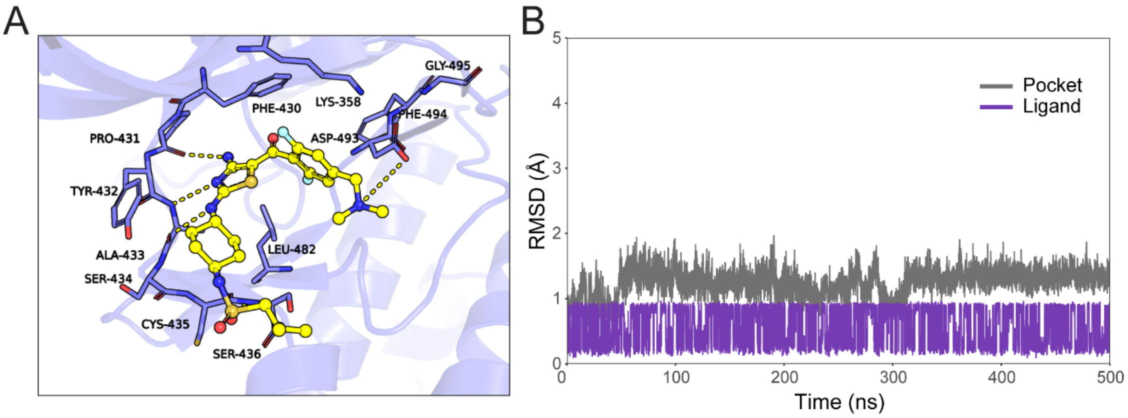

Figure S3. The binding conformation of Cmpd2 in the *T. brucei* CRK12 pocket and the overall structural stability of the CRK12-Cmpd2 complex. (A) Docking pose of Cmpd2 with *T. brucei* CRK12. (B) The RMSD of the heavy atoms of the binding pocket and the ligand Cmpd2 in the CRK12-Cmpd2 complex.

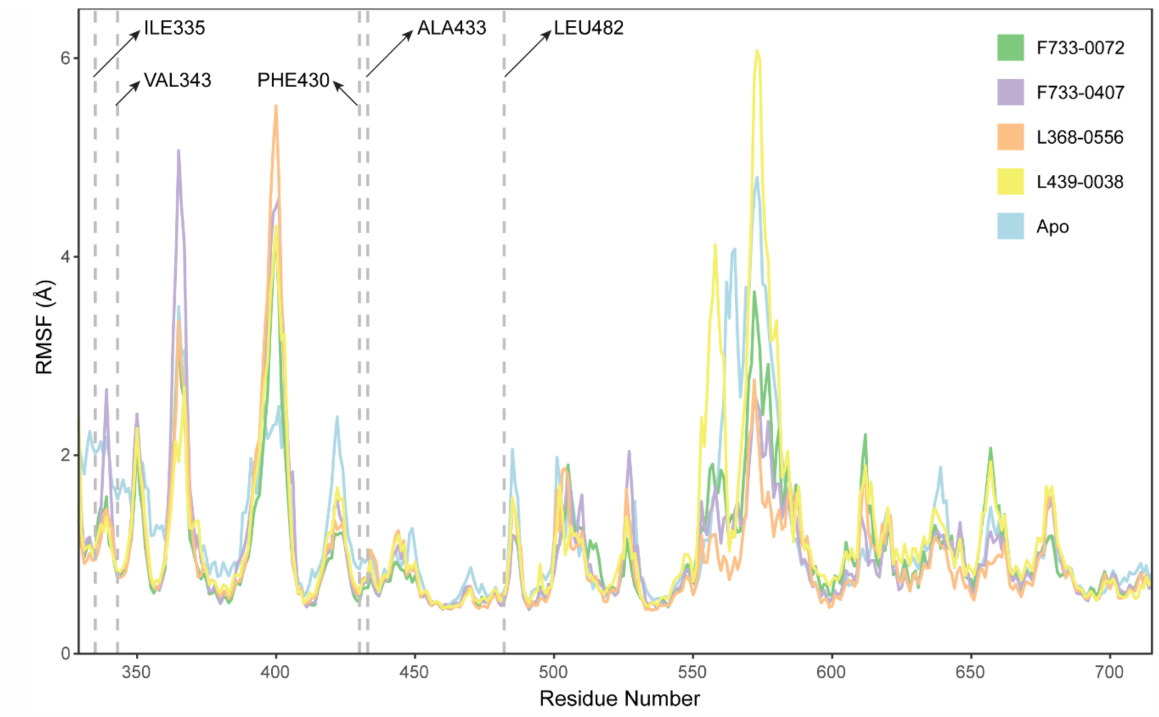

Figure S4. Root Mean Square Fluctuation (RMSF) of Cα atoms in CRK12. Key hotspot residues involved in ligand binding are annotated with dashed vertical lines.

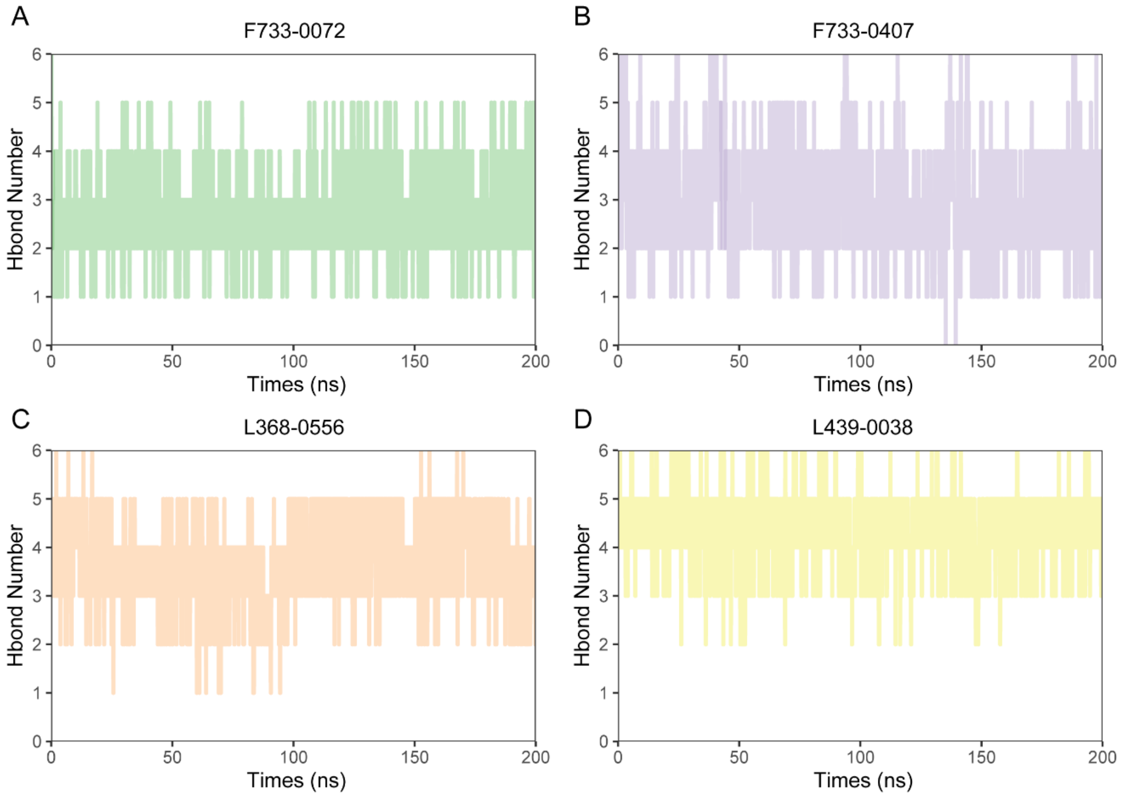

Figure S5. The monitoring of the total number of hydrogen bonds between CRK12 and four hit compounds (**A**) F733-0072, (**B**) F733-0407, (**C**) L368-0556, and (**D**) L439-0038 during 200 ns MD simulations.

Table S1. Physicochemical properties of the compounds derived from the virtual screening.

Compound ID	MW	logP	donorHB	accptHB	PSA	Glide gscore	Glide Docking score	Prime MMGBSA
F733-0072	458.61	3.98	2	9	79.28	-10.79	-10.70	-86.93
F145-0373	376.47	0.35	3	8.5	130.81	-10.67	-10.67	-96.12
F733-0407	433.53	4.26	2	7	76.83	-10.41	-10.41	-90.30
SA99-0102	390.87	2.12	3	8.2	109.50	-10.60	-10.19	-88.54
L439-0038	457.53	5.19	2	7	110.59	-10.47	-10.47	-89.48
E139-0046	370.79	3.44	1	5.5	70.16	-9.89	-9.88	-79.31
E139-0048	400.82	3.42	1	6.25	77.49	-9.80	-9.79	-81.10
E140-0031	470.97	3.04	2	8.75	96.91	-10.42	-9.76	-94.43
G490-0575	431.32	3.95	2	8.5	91.05	-10.20	-9.72	-89.99
G194-0302	381.43	1.33	0.25	6	75.21	-9.60	-9.60	-86.47
Y205-7152	402.50	2.30	1	7.5	95.65	-9.37	-9.33	-90.08
S564-0505	405.50	3.35	2	5.25	87.29	-9.47	-9.29	-84.59
3214-0082	338.19	4.62	2	3	68.37	-9.24	-9.23	-63.97
Y041-7264	358.34	3.50	1	2.75	69.41	-9.20	-9.20	-78.36
L368-0556	421.58	4.19	2	7	75.53	-9.10	-9.10	-77.60
SC15-0275	447.54	2.14	1	8	100.36	-9.05	-9.05	-97.67
C926-0334	435.44	2.93	2	7.75	136.91	-8.92	-8.92	-68.19
P500-0561	415.89	3.13	1	6.5	67.87	-8.89	-8.89	-79.81
F109-0400	362.39	4.36	1	5.5	75.68	-8.87	-8.87	-76.87
Y044-4665	388.44	1.73	2	8.25	126.28	-8.82	-8.82	-84.22
3810-0403	463.51	4.94	1	6	109.11	-8.84	-8.81	-93.73
L934-0598	414.50	2.11	1	8.5	106.89	-8.79	-8.60	-88.42
L872-0038	444.55	3.66	1	7.5	74.83	-9.10	-8.58	-96.87
E687-0429	464.52	2.75	1	9.25	112.20	-8.95	-8.54	-90.94
G346-0042	470.56	4.21	1	7.5	95.42	-8.44	-8.43	-92.82
F127-0390	407.49	3.61	2	7.5	104.48	-8.36	-8.36	-80.70

Table S2. The coordinates (Å) and RESP atomic charges for the optimized geometry of ligand Cmpd2 obtained at the HF/6-31G level.

Atom id	Atom name	x	y	z	Charge
1	C1	8.046	3.285	-0.313	-0.63748
2	C2	6.534	3.036	-0.321	0.49452
3	C3	5.951	3.071	1.095	-0.63748
4	C4	6.137	1.792	-1.139	-0.165101
5	S1	6.466	0.164	-0.479	1.227337
6	O1	6.298	-0.745	-1.575	-0.600664
7	O2	7.686	0.183	0.272	-0.600664
8	N1	5.254	-0.124	0.612	-0.801504
9	C5	4.255	-1.172	0.353	0.349686
10	C6	3.624	-1.563	1.691	-0.123731
11	C7	2.538	-2.626	1.508	-0.165425
12	C8	1.467	-2.172	0.514	0.31745
13	N2	0.49	-3.239	0.343	-0.771674
14	C9	-0.817	-3.122	0.132	0.786047
15	N3	-1.559	-4.161	-0.103	-0.800106
16	C10	-2.857	-3.813	-0.26	0.958495
17	N4	-3.743	-4.766	-0.503	-1.05845
18	C11	-3.157	-2.451	-0.151	-0.586061
19	C12	-4.451	-1.904	-0.278	0.679843
20	O3	-5.45	-2.546	-0.518	-0.603122
21	C13	-4.639	-0.402	-0.118	-0.234902
22	C14	-4.946	0.394	-1.209	0.471828
23	F1	-4.929	-0.141	-2.41	-0.180887
24	C15	-5.27	1.729	-1.096	-0.494728
25	C16	-5.292	2.31	0.167	0.195664
26	C17	-5.669	3.761	0.306	-0.132553
27	N5	-4.553	4.686	-0.139	0.013439
28	C18	-5.043	6.084	-0.324	-0.395698

Atom id	Atom name	x	y	z	Charge
29	C19	-3.368	4.641	0.769	-0.395698
30	C20	-4.989	1.551	1.284	-0.494728
31	C21	-4.675	0.214	1.116	0.471828
32	F2	-4.406	-0.497	2.192	-0.180887
33	S2	-1.646	-1.581	0.172	-0.230082
34	C22	2.098	-1.754	-0.819	-0.165425
35	C23	3.178	-0.687	-0.618	-0.123731
36	H1	8.576	2.513	0.228	0.15934
37	H2	8.259	4.24	0.157	0.15934
38	H3	8.441	3.321	-1.324	0.15934
39	H4	6.078	3.854	-0.876	-0.039309
40	H5	6.426	2.339	1.738	0.15934
41	H6	6.12	4.048	1.535	0.15934
42	H7	4.882	2.882	1.097	0.15934
43	H8	5.077	1.792	-1.358	0.058127
44	H9	6.663	1.787	-2.086	0.058127
45	H10	5.669	-0.184	1.521	0.386421
46	H11	4.737	-2.044	-0.073	0.047087
47	H12	4.385	-1.943	2.366	0.042121
48	H13	3.198	-0.676	2.158	0.042121
49	H14	2.994	-3.545	1.143	0.076315
50	H15	2.077	-2.859	2.461	0.076315
51	H16	0.946	-1.323	0.944	0.058266
52	H17	0.827	-4.174	0.249	0.414862
53	H18	-4.703	-4.53	-0.627	0.478815
54	H19	-3.436	-5.71	-0.561	0.478815
55	H20	-5.538	2.269	-1.987	0.225091
56	H21	-6.521	4	-0.314	0.154733
57	H22	-5.899	4.029	1.327	0.154733
58	H23	-5.411	6.448	0.624	0.194817

Atom id	Atom name	x	y	z	Charge
59	H24	-4.226	6.702	-0.664	0.194817
60	H25	-5.837	6.087	-1.055	0.194817
61	H26	-3.665	5.015	1.737	0.194817
62	H27	-3.023	3.624	0.85	0.194817
63	H28	-2.591	5.266	0.354	0.194817
64	H29	-5.02	1.954	2.28	0.225091
65	H30	1.329	-1.387	-1.491	0.076315
66	H31	2.534	-2.631	-1.291	0.076315
67	H32	2.736	0.225	-0.22	0.042121
68	H33	3.624	-0.445	-1.574	0.042121
69	H34	-4.246	4.35	-1.04	0.315098
